# Giant dedifferentiated liposarcoma of small bowel mesentery: a case report

**DOI:** 10.1186/s12957-016-1007-1

**Published:** 2016-09-21

**Authors:** Susanta Meher, Tushar Subhadarshan Mishra, Satyajit Rath, Prakash Kumar Sasmal, Pritinanda Mishra, Susama Patra

**Affiliations:** 1 Department of General Surgery, All India Institute of Medical Sciences, Room no. 403, Academic Block, Sijua, Bhubaneswar, Odisha 751019 India; 2 Department of Pathology, All India Institute of Medical Sciences, Sijua, Bhubaneswar, Odisha 751019 India

**Keywords:** Dedifferentiated liposarcoma, Small bowel, Mesentery

## Abstract

**Background:**

Dedifferentiated liposarcoma is an uncommon variant of liposarcoma, with poor prognosis and higher preponderance to local recurrence. Only nine cases of dedifferentiated liposarcoma of small bowel mesentery have been reported till now. This is a case of giant dedifferentiated liposarcoma of the small bowel mesentery, weighing nearly 9 kg (19.8 lbs), with synchronous lesions in the extraperitoneal space, which is the first such case to be reported.

**Case presentation:**

We report a case of a 62-year-old man, who presented with a huge abdominal mass occupying nearly the entire abdomen. A contrast enhanced computed tomography of abdomen and pelvis revealed a large, poorly enhancing, heterogeneous, lobulated mass of size 27 × 16 cm, displacing the bowel loops peripherally. At laparotomy, a large mass arising from the small bowel mesentery was found. In addition, many other smaller synchronous lesions were studded in the entire small bowel mesentery and a couple more in the extraperitoneal space. A palliative excision of the giant mass along with the adjacent small bowel was done. The other smaller swellings were not causing any mass effect and were left behind as they were numerous, virtually ruling out any possibility of a curative excision. The histopathological examination suggested the diagnosis of dedifferentiated liposarcoma. On immunohistochemistry, S-100 was positive in the well-differentiated sarcomatous areas. The CD 117 and SMA were strongly negative ruling out the possibility of a gastrointestinal stromal tumour. The CD 34 however was positive in the tumour cells.

**Conclusions:**

Dedifferentiated liposarcoma of the small bowel mesentery is rare. Involvement of nearly whole of the small bowel mesentery in the disease process virtually rules out the possibility of a curative resection, the mainstay of management. This report would add to the knowledge of this rare disease and the possible therapeutic problem that may be encountered in case of multifocal disease.

## Background

Liposarcoma is the most common soft tissue sarcoma in adults [1]. Evans, in 1979, was the first to identify the dedifferentiated variant of liposarcoma [2]. Dedifferentiated liposarcomas (DDLPS) have features of well-differentiated as well as poorly differentiated liposarcoma along with nonlipomatous sarcoma in the same tumour. The prognosis of DDLPS variant of liposarcoma is worse than the well-differentiated ones, but better than the pleomorphic type. The retroperitoneum is the most frequent site of its occurrence [1, 2]. DDLPS affecting the small bowel mesentery is a rarity [2]. This case of dedifferentiated liposarcoma of small bowel mesentery is being reported for the therapeutic problem posed by the presence of a large number of nodules in the mesentery as well as a few in the extraperitoneal space. Multiple DDLPS of the small bowel mesentery with synchronous lesions in the extraperitoneal space is the first such case to be reported, to the best of our knowledge.

## Case presentation

A 62-year-old man presented to us with history of a palpable abdominal mass, vague left side abdominal pain and loss of appetite for 2 months. The bowel habits were normal, and there was no history of weight loss. He was a known hypertensive on medications. On examination, the patient was thin built and had a huge abdominal mass extending from the epigastrium to the hypogastrium including both the flanks.

**Fig. 1:**
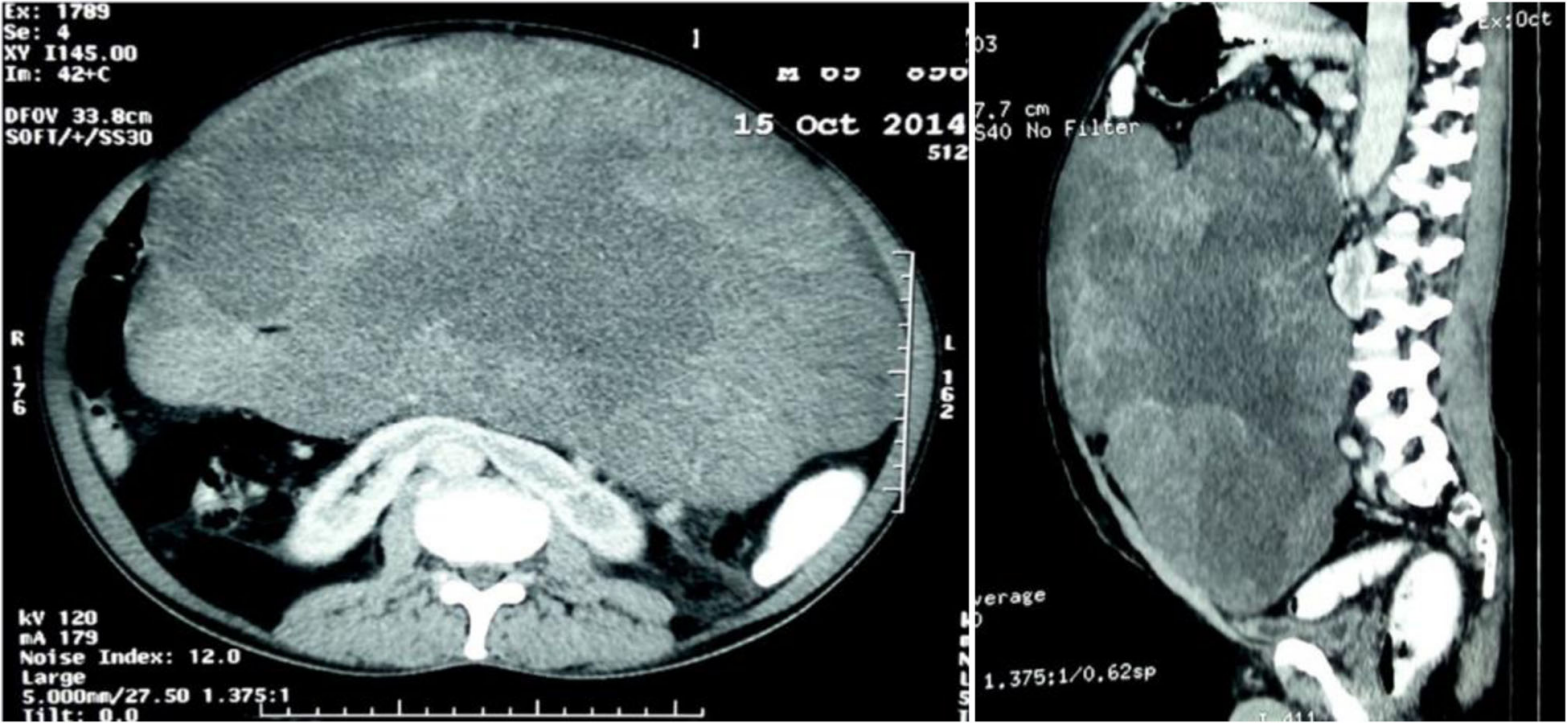
CECT abdomen and pelvis, showing a large poorly enhancing heterogeneous mass

The routine hematogical and biochemical parameters were within normal limits. The tumour markers including CA19-9, carcinoembryonic antigen (CEA) and CA-125 levels were also within normal limits. Abdominal sonography revealed a heterogeneous, nodular, soft tissue mass nearly filling the abdominal cavity. Computed tomography showed a large, poorly enhancing, lobulated soft tissue mass of size 27 × 16 cm with fluid densities. The mass was displacing the bowel loops peripherally (Fig. 1). There was no evidence of calcification or ascites.

**Fig. 2:**
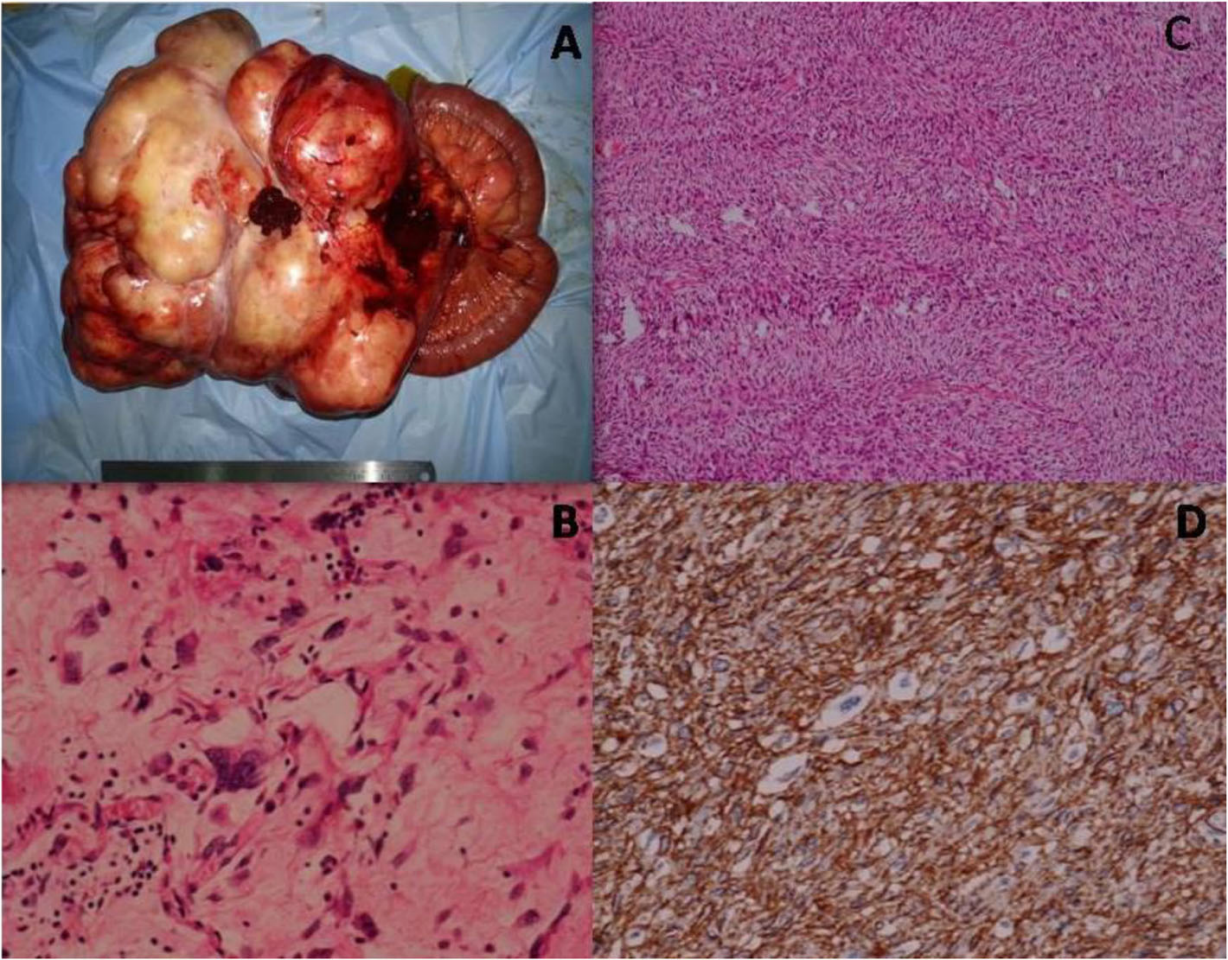
**a** Post operative specimen showing a huge, well-circumscribed lobulated mass with multiple mesenteric nodules. **b** The well-differentiated areas showing lipoblasts. **c** The nonlipomatous areas showing spindle cells, arranged in long and short fascicles and in storiform pattern. **d** Tumour cell showing CD 34 positivity

On laparotomy, a huge well-circumscribed lobulated mass was identified arising from the jejunal mesentery 20 cm away from the ligament of Trietz. Multiple nodular lesions were found adjacent to the main tumour mass, largest measuring 3.5 × 2.5 × 3 cm (Fig. 2). There were two extraperitoneal nodules measuring 2.5 cm × 2.5 cm in size in maximum dimensions, present one at the right deep inguinal ring, and the other near the right iliac vessels. Segmental resection of the small bowel was done including the tumour mass and the mesenteric nodules.

Gross examination revealed a large lobulated mass of size 35 × 25 × 25 cm, weighing 9 kg (19.8 lbs). Cut section showed a greyish yellow, soft to firm tumour with solid components along with myxoid area in less than 50 % area. The attached small bowel with mesentery measured 40 cm. There were multiple nodules over the mesentery, largest measuring 3.5 × 2.5 × 3 cm and smallest measuring 0.5 × 0.5 cm.

Histopathological examination showed a partially encapsulated tumour composed of spindle cells, arranged in long and short fascicles and in storiform pattern. The neoplastic cells were oval to spindloid with round to oval pleomorphic hyperchromatic nuclei, with prominent nucleoli. Binucleated and multinucleated giant cells were seen. Round cells with nucleus pushed laterally with vacuolated cytoplasm resembling lipoblasts were present interspersed in between the tumour cells. There was a zone of transition between the well-differentiated area to a high-grade sarcomatous area with extensive coagulative necrosis, hyalinisation and myxoid changes. Mononuclear cell infiltration including lymphocytes and plasma cells were present. Mitotic rate was 8–10/10 per high power field. A diagnosis of dedifferentiated liposarcoma was made based on the basis of histological features. On immunohistochemistry, S-100 was positive in the well-differentiated sarcomatous areas. The CD 117 and SMA were strongly negative ruling out the possibility of a gastrointestinal stromal tumour (GIST). The CD 34 however was positive in the tumour cells. In the first follow-up after 1 month, the patient had gained weight and had regained his appetite. He had a general overall feeling of well-being. There was no significant problem at 10 months post surgery.

### Discussion

According to the WHO, lipomatous tumours are classified into benign, intermediate and malignant types. The well-differentiated liposarcoma belongs to the intermediate variety being locally aggressive. The malignant type includes the dedifferentiated, myxoid, round cell, pleomorphic, mixed and liposarcoma not otherwise specified types. The dedifferentiated variant of liposarcoma is thrice more common in the retroperitoneum than in the extremities, where well-differentiated liposarcomas are more usual [1]. DDLPS primarily arising from the gastrointestinal tract or its secondary involvement is very unusual [2, 3]. DDLPS involving small bowel mesentery is a rare entity, with nine such cases having been described in the literature as of now (Table 1).

**Table 1:** Dedifferentiated liposarcoma of small bowel mesentery reported in the literature

Reference	Age (in years)/gender	Multifocality	Maximum size (in cm)	Primary treatment	Adjuvant therapy
Hasegawa et al. [10]	59/M	Unifocal	14	Surgery	No
Hasegawa et al*.* [10]	58/F	Unifocal	20	Surgery	No
Hasegawa et al. [10]	56/F	Unifocal	20	Surgery	No
Hasegawa et al. [10]	52/F	Unifocal	40	Surgery	No
Hasegawa et al. [10]	63/F	Unifocal	10	Surgery	No
Korukluoglu et al. [11]	61/F	Bifocal	25 and 16	Surgery	Chemotherapy
Grifasi et al. [4]	59/F	Unifocal	25	Surgery	Ifosfamide (on recurrence)
Cha et al. [2]	76/F	Unifocal	5	Surgery	No
Vats M [12]	36/F	Multifocal	25	Surgery	Chemotherapy (doxorubicin, dacarbazine, ifosfamide)
Present case	62/M	Multifocal	35	Surgery	No

The early symptoms of this disease are nonspecific; they present as abdominal lumps when they attain large sizes and may cause pressure symptoms [2, 3]. X-ray, ultrasonography, computed tomography and MRI can be used for radiological assessment of the tumour [4]. Mesenteric angiography has been used to ascertain the location of the tumour preoperatively [5]. Needle biopsy can be fallacious due to inadequate sampling, making it difficult to distinguish from high-grade sarcomas [2, 3]. Thorough sampling from different sites of the tumour is mandatory as it is as important to identify the nonlipogenic component as it to see the well-differentiated areas to reach at the diagnosis [3]. Hence, post operative histopathology is the only reliable method of stamping the diagnosis of DDLPS. Histological DDLPS is characterised by a mixture of atypical lipoma (ALT)/well-differentiated liposarcoma (WDLPS) and a high-grade nonlipogenic sarcomatous component, usually with an abrupt transition between the two components [1–3]. Dedifferentiated areas exhibit a wide morphological spectrum and often show a high-grade sarcoma resembling a malignant fibrous histiocytoma (MFH) or low-grade spindle cell sarcoma [1–3].

Distinction of different subtypes of liposarcoma on histopathological examination and immunohistochemistry is important because DDLPS is the variant of liposarcoma having worse prognosis than the well-differentiated variety. Its clinical course however is less aggressive than other types of high-grade sarcoma. [1–3]. Due to its proximity to the bowel, it becomes imperative to distinguish DDLPS from GIST. CD117 (c-kit) and CD34 which are expressed in GIST are usually negative in DDLPS [2, 3]. In our case, CD117 and SMA were negative. The CD 34 however was positive in our case. It is worth emphasising that CD 34 expression is not specific to GIST, and it may also be positive in tumours like solitary fibrous tumour, inflammatory fibrous polyps and dedifferentiated liposarcoma [6]. MDM2 amplification demonstrated by florescent in situ hybridisation (FISH) technique, immunohistochemistry, and quantitative PCR help in the diagnosis of lipomatous tumours. Hence, it may have a role in differentiating these tumours from the poorly differentiated sarcomas [4]. p53 has a tumour suppression role. Mutations of this gene therefore are associated with carcinogenesis and tumours with poor prognosis. Karaman et al. found p53 gene mutation in nonlipomatous component of dedifferentiated sarcomas and not in the well-differentiated areas [7].

Complete surgical resection is the only effective treatment option available [4]. Efforts to remove the tumour completely often require the removal of an organ or part of an organ to which it is adherent [3, 4]. Even palliative resection is sometimes helpful to treat troublesome symptoms of recurrence [5, 8]. The negative margins of surgical resection are associated with disease-free survival and overall survival [4]. Although the evidence supporting the use of chemotherapy and radiotherapy is limited [3, 4], they may be of benefit in poorly differentiated sarcomas more than 10 cm in size or if there is residual disease after a palliative surgery [9]. Ifosfamide in high doses has been tried in recurrent DDLPS, after recurrence [4]. However, approximately 40 % of DDLPS will have local recurrence, 17 % will metastasize and 28 % will have tumour-related mortality [1].

## Conclusions

Dedifferentiated liposarcoma is a variant of liposarcoma having worse prognosis and higher risk of local recurrence than the well-differentiated variety. Hence, accurate histopathological diagnosis is essential for prognostication and closer follow-up. Complete surgical excision with negative margin is associated with overall survival and disease-free survival. However, DDLPS at multiple locations in the mesentery is a possibility which may exclude the chance of curative resection. Palliative resection upfront, of large offending masses, or upon recurrence will have the benefit by relieving pressure symptoms due to the tumour. The scope of adjuvant chemotherapy and radiotherapy is limited to recurrent, residual or poorly differentiated liposarcomas.

## Abbreviations

ALT: Atypical lipoma
CEA: Carcinoembryonic antigen
DDLPS: Dedifferentiated liposarcomas
FISH: Fluorescence in situ hybridization
GIST: Gastrointestinal stromal tumour
MFH: Malignant fibrous histiocytoma
WDLPS: Well-differentiated liposarcomas
